# What Is the Optimal Therapy for Patients with H5N1 Influenza?

**DOI:** 10.1371/journal.pmed.1000091

**Published:** 2009-06-23

**Authors:** Nicholas J. White, Robert G. Webster, Elena A. Govorkova, Timothy M. Uyeki

**Affiliations:** 1 Faculty of Tropical Medicine, Mahidol University, Bangkok, Thailand; 2 Department of Infectious Diseases, Division of Virology, St. Jude Children's Research Hospital, Memphis, Tennessee, United States of America; 3 Epidemiology and Prevention Branch, Influenza Division, National Center for Immunization and Respiratory Diseases, Centers for Disease Control and Prevention, Atlanta, Georgia, United States of America

## Abstract

In a 2007 article in *PLoS Medicine* [**10**], Holger J. Schünemann and colleagues described a new process used by the World Health Organization for rapidly developing clinical management guidelines in emergency situations. These situations include outbreaks of emerging infectious diseases. The authors discussed how they developed such a “rapid advice” guideline for the pharmacological management of avian influenza A (H5N1) virus infection. The guideline recommends giving the antiviral drug oseltamivir at a dose of 75 mg twice daily for five days. In this Debate, Nicholas White argues that such dosing is inadequate, Robert Webster and Elena Govorkova say that combination antiviral therapy should be used, and Tim Uyeki reminds us that clinical care of patients with H5N1 entails much more than antiviral treatment. These issues may also apply to therapy of patients hospitalized with severe disease due to novel swine-origin influenza A (H1N1) virus infection.

## Abstract 2

Nicholas White discusses optimal dosing of oseltamivir, Robert Webster and Elena Govorkova discuss combination antiviral therapy, and Timothy Uyeki discusses clinical care of patients with H5N1.

## Nicholas White's Viewpoint: Common Sense Argues That High Doses of Oseltamivir Should Be Used

### Developing Treatment Guidelines for Potentially Lethal Infections

Rapidly fatal infections need urgent treatment with optimum doses of appropriate antimicrobials. Such doses should ideally produce maximum effects as quickly as possible, and provide the greatest differential between lives saved and lives lost because of toxicity. If the antimicrobial drug is not eliminated rapidly, a loading dose should be given to provide therapeutic concentrations as soon as possible.

This dosing strategy for rapidly fatal infections contrasts with dose recommendations for uncomplicated infections—such recommendations are aimed at lower microorganism burdens, where rapidity of action is less important and adverse effects are of greater significance. In other words the risk-benefit trade-off, commonly termed the therapeutic ratio, is different in severe and uncomplicated infections.

This difference has important implications for treatment guidelines. According to the World Health Organization (WHO): “Guidelines are formal advisory statements which should be robust enough to meet the unique circumstances and constraints of the specific situation to which they are being applied” [1]. Treatment guidelines are best when they rest on a sound and consistent evidence base [1],[2]. Randomised clinical trials (RCTs) are considered to provide the best evidence. But what if the evidence from controlled trials is insufficient, or there simply isn't any? Guidance and specific recommendations are still necessary. Inadequate initial treatment of life-threatening infections has serious consequences. Therefore, common sense argues for recommending higher doses for such infections, at the expense of increased toxicity, to avoid any possibility of under-dosing those patients with unusual pharmacokinetics and more resistant organisms. If intravenous administration is not possible, absorption from the gut or intramuscular injection site may be compromised in the most seriously ill, arguing again for higher doses.

In this context of critical uncertainty, and against a background of concerns over liability and consequent risk aversion, physicians often seem more worried about the risks of adverse effects than of under-dosing, even though antimicrobial adverse effects are rarely fatal. Seldom is an infectious disease death ascribed to administration of inadequate doses.

### Treatment Guidelines for H5N1 Influenza

H5N1 influenza is regarded by many as the greatest threat to human health and national security [3]. Fortunately human infections are still rare, but this rarity also means that there are no published RCTs of treatment. The oral viral neuraminidase inhibitor oseltamivir (Tamiflu) is considered the drug of choice [4],[5]. There is no parenteral formulation. H5N1 influenza replicates more rapidly than seasonal influenza viruses [4],[5], reaching much greater viral burdens than do other human influenza viruses [6]. Resistance arises readily. Mortality consistently exceeds 50%, which puts H5N1 influenza amongst the most lethal of human infections.

Experimental studies with H5N1 viruses in animal models suggest that high doses and long courses of neuraminidase inhibitors provide optimal treatment [7],[8]. Despite this evidence, a “rigorous and transparent” process, led by WHO, to develop treatment guidelines for H5N1 influenza has recommended an adult dose of 75 mg twice daily for five days. This is the “standard” dose regimen for uncomplicated seasonal influenza [9],[10]. If absorbed well, this 75 mg dose might provide maximal neuraminidase inhibition at the sites of infection in all patients seriously ill with H5N1 influenza. In other words it might be enough, but the truth is that we just do not know. The concentration-effect relationship in patients has not been characterised. Oseltamivir doses of up to 1,000 mg have been given to volunteers. High doses of oseltamivir are reasonably well tolerated in humans, and there is experimental evidence to suggest they could be more effective [5],[7],[8],[11],[12].

There seems little to gain and everything to lose by using a low dose of this potentially life-saving drug in a highly lethal infection. The “evidence-based approach” (Grading of Recommendations Assessment, Development and Evaluation or GRADE; see http://www.gradeworkinggroup.org/), now considered “state of the art” for guideline development [1],[13],[14], has been constrained by lack of RCT evidence on higher doses of oseltamivir. In recent years a hierarchy of the quality of evidence has been increasingly promoted, particularly for the formulation of guidelines. “Hierarchies place randomised controlled trials (RCTs) at their summit, with various forms of observational studies nestling in the foothills,” says Rawlins [15], but information from observational studies and other foothill inhabitants (experimental investigations, analogy with similar conditions and processes, pathological and pharmacological understanding and reasoning, and a derived assessment of risks and benefits) is also valuable. In the case of pandemic influenza, the GRADE process has resulted in a dose recommendation for H5N1 influenza that could be too low.

### Using All the Evidence To Assess the Risks and Benefits

In contrast to the GRADE approach, a “mechanism-based” approach, incorporating current understanding of this lethal disease and of antimicrobial pharmacology, and assessing the risks and benefits, would lead to initial use of the highest oseltamivir doses considered to have a low risk of major toxicity in H5N1 influenza. This fundamental difference in analytical and deductive approaches is analogous to the frequentist versus Bayesian debate in statistics. Rawlins has recently articulated the important limitations of relying too much upon evidence from RCTs and has argued cogently for greater use of a Bayesian approach in decision making on recommendations for therapeutic interventions [15]. Different approaches to the same problem may yield different results initially, although as evidence accrues, results of the two approaches tend to converge.

The current approach to guideline development may be too restrictive. Where there is little or no direct evidence from RCTs, the current “evidence-based approach” to treatment guidelines certainly needs reconsideration. In the absence of direct evidence on dosing in a rapidly lethal infection, basic precepts of antimicrobial pharmacology (“Bayesian priors”) and common sense argue that the highest possible dose of an antimicrobial should be used, at least initially, until evidence becomes available to inform the recommendation.

## Robert Webster and Elena Govorkova's Viewpoint: Effective H5N1 Influenza Management Calls for the Adoption of a Multidrug Approach

Nature has again sent a message to scientists and public health officials concerned with the current pharmacological treatment of humans with a potential pandemic influenza virus. The message is loud and clear that the strategy of relying on single anti-influenza drug treatment is wrong. The rapid emergence of seasonal influenza A (H1N1) viruses resistant to oseltamivir in Scandinavia at the end of 2007 (where little or no anti-influenza drugs are used) was unexpected [16]. These resistant viruses contain the His274Tyr neuraminidase mutation and have remarkable fitness; they spread globally in less than a year [17]. While the oseltamivir-resistant influenza A (H1N1) viruses are still susceptible to the neuraminidase inhibitor zanamivir (Relenza), it would be foolish to continue being complacent and rely on monotherapy. Influenza viruses have a segmented RNA genome that is error-prone during replication and lacks proofreading mechanisms. This fundamental property of influenza viruses guarantees that resistant variants will emerge. Such resistance may occur spontaneously and naturally (without drug intervention) but would be facilitated by the use of single-agent chemotherapy with oseltamivir alone.

Extensive experience treating human immunodeficiency virus (HIV) clearly showed the futility of single-agent antiviral therapy; drug-resistant HIV strains emerged almost immediately in patients receiving monotherapy [18],[19]. The subsequent, successful management of HIV with multidrug combinations of highly active antiretroviral therapy (HAART) has enabled thousands of patients to control their disease and live productive lives. HAART targets multiple functions of the virus (i.e., reverse transcription, protein synthesis, attachment, and entry) [20]. These lessons from HIV must be applied to influenza.

The reason given for continuation of anti-influenza monotherapy is that drugs targeting specific viral functions are not available; this partial truth is simply an excuse that impedes our progress. Currently, two classes of anti-influenza drugs are available: the adamantanes (amantadine and rimantadine) and the neuraminidase inhibitors (oseltamivir and zanamivir). Amantadine resistance predominates among seasonal influenza A (H3N2) viruses, and the ineffectiveness of the adamantanes against influenza B viruses makes their use pointless. In fact, their use is counterproductive, because it facilitates selection of resistance.

Can we use available therapies to minimize the impact of H5N1 influenza outbreaks in humans? There are at least ten clades of H5N1 influenza viruses [21]. Two dominant clades affect humans: clades 1 and 2.1 viruses are often adamantane-resistant, and representatives of clades 2.2 and 2.3 are adamantane-susceptible [4]. Clades 1 and 2 are susceptible to neuraminidase inhibitors, and the current WHO guidelines suggest that clinicians administer a double dose of oseltamivir to severely ill patients because of the drug's uncertain absorption and the high disease mortality [22]. However, given the ease with which naturally occurring oseltamivir-resistant H1N1 viruses emerge and become dominant, N1 neuraminidase and H5N1 viruses will probably have a similar fate. Thus, we must consider a multidrug approach to managing patients with H5N1.

Combination chemotherapy for influenza is supported by data from animal models. Combinations of oseltamivir and amantadine inhibited H5N1 virus replication in the lungs and brains of infected mice, whereas monotherapy was only partially effective [23]. The combination of oseltamivir and ribavirin (a polymerase inhibitor, although not approved for influenza in most countries) showed additive efficacy against clades 1 and 2, though efficacy differences were seen against two different strains [24]. The combination of oseltamivir and ribavirin is far from optimal, but many approaches to combination therapy for influenza are in the pipeline: (1) development of additional neuraminidase inhibitors or parenteral drug formulations; (2) new antiviral targets, including the polymerase and hemagglutinin molecule and attachment inhibition; (3) modulation of overexuberant innate host response; (4) antibody-mediated therapy; and (5) combined antiviral and vaccine strategies. Currently under development is the neuraminidase inhibitor peramivir, which has three chemical groups that interact with the active-site residues of neuraminidase, resulting in tight binding and a slow rate of dissociation.

The development of intravenous and intramuscular drug formulations will also provide advantages against systemically replicating H5N1 influenza viruses. Long-acting, single-dose inhaled neuraminidase inhibitors will probably be available in a few years. Other potential targets for drug development include the surface protein hemagglutinin and polymerase inhibitors. T-705, a potent inhibitor of viral RNA polymerase, is active against neuraminidase inhibitor-resistant and amantadine-resistant viruses. New treatments such as immunomodulatory drugs that potentially control immune system-mediated tissue damage will require strong experimental evidence before adoption.

Combination chemotherapy consisting of anti-influenza drugs and inflammation inhibitors (e.g., celecoxib and mesalazine) was recently reported as a promising approach to control H5N1 infection in mice [25]. Another exciting recent discovery is the ability of the type II diabetes drug pioglitazone to modulate tissue-damaging compounds of the innate immune response without compromising T cell-mediated viral clearance [26]. The passive administration of humanized monoclonal antibodies to H5 hemagglutinin in mice [27],[28] and the creation of comprehensive influenza antibody libraries from survivors of the H5N1 avian influenza [29] have also provided encouraging data.

**Figure 1:**
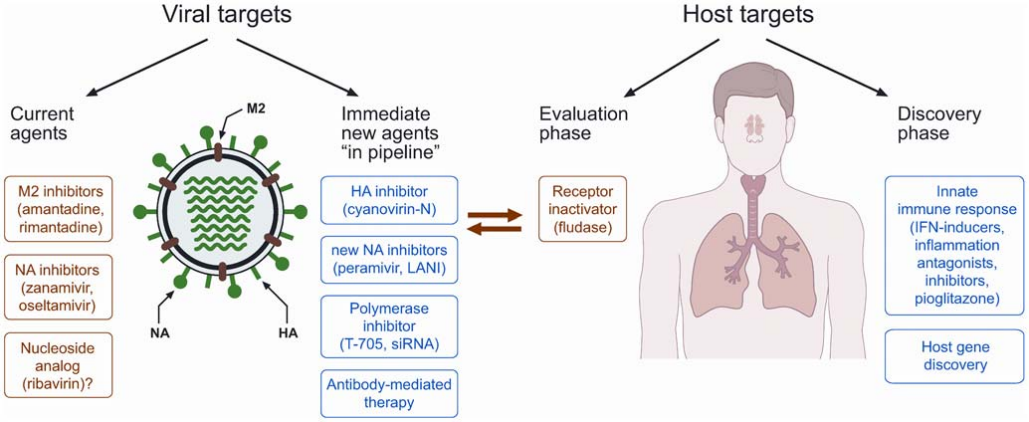
A multidrug approach to the management of influenza. HA, hemagglutinin; IFN, interferon; LANI, long-acting neuraminidase inhibitor; NA, neuraminidase; siRNA, small interfering RNA.

Many virus and host factors influence the outcome of H5N1 disease progression. Therefore multiple anti-influenza drugs must be used to effectively treat and prevent the spread of infection (Figure 1). Results from our experiments [23],[24] suggest that a combination therapy approach guards against the emergence of resistant strains. Thus, we propose that combinations of adamantanes and neuraminidase inhibitors be immediately introduced, ribavirin use be further evaluated, and clinical trials of T-705 proceed with urgency.

## Tim Uyeki's Viewpoint: Clinical Management of Patients with H5N1 Is Challenging—Data Are Needed To Guide Clinicians

The emergence of a new respiratory infection that can cause rapid severe outcomes, including death, presents major challenges for identifying optimal clinical management and treatment. For example, early in the severe acute respiratory syndrome (SARS) epidemic, oral or intravenous ribavirin was administered to patients with SARS on the assumption that this antiviral medication would have activity against a suspected respiratory viral pathogen. Unfortunately, it is unclear if ribavirin was beneficial for treatment of patients with SARS, and hemolytic anemia occurred in some treated patients [30],[31]. Methylprednisolone was also administered widely to patients with SARS, and higher doses were associated with avascular necrosis among survivors [32],[33]. To date, no definitive treatment exists for human infection with SARS-associated coronavirus (SARS-CoV), and there are still challenges in collecting data from controlled clinical treatment trials of novel pathogens [34].

Since 1997, sporadic human infection with highly pathogenic avian influenza A (HPAI) H5N1 virus has caused illness in more than 440 persons in 15 countries [35]–[37], with mortality consistently higher than 60% among reported cases [37]. In contrast with the emergence of human infection with SARS-CoV, a pathogen with no previously documented treatment, HPAI H5N1 virus is an influenza A virus, and antiviral medications with documented in vitro activity and clinical benefit for related susceptible seasonal influenza A subtype viruses have existed for years. However, most published clinical studies of antiviral treatment for human infection with seasonal influenza A viruses have been conducted among otherwise healthy outpatients with uncomplicated influenza in which early treatment (less than 48 hours from illness onset) was assessed [38]–[41]. There have been only two published retrospective studies of treatment among hospitalized elderly [42],[43].

In the absence of controlled clinical treatment trial data for patients with HPAI H5N1 virus infection, what is the best guidance based upon available data? Is it valid to extrapolate data from early treatment of seasonal influenza A virus infection, largely for uncomplicated influenza among outpatients, to hospitalized patients with severe HPAI H5N1 disease, especially when the pathogenesis may be different [4],[6]? In 2006, WHO convened a panel to assess the available evidence and issued guidance for antiviral treatment and chemoprophylaxis of H5N1 virus infection [9],[10]. Limited data suggest higher H5N1 patient survival with earlier or any oseltamivir treatment (standard dosing for five days) compared to late or no treatment [4],[44],[45]. WHO also issued guidance recommending consideration of higher oseltamivir dosing and longer duration of treatment, especially for patients with late clinical presentation and severe disease [4],[22], and recommended against routine corticosteroid treatment [4],[22]. To date, clinical data on H5N1 antiviral treatment to guide clinicians are limited.

Multiple challenges confront the clinician caring for a patient with suspected or confirmed H5N1 virus infection. First, HPAI H5N1 virus is dynamic and a “moving target.” H5N1 virus strains continue to evolve into multiple genotypes and antigenically distinct clades and subclades, and at least ten clades of H5N1 virus strains have been identified to date [4]. Virus strains in four clades, including three subclades of clade 2 viruses, have infected humans to date [4].

Furthermore, these strains have different in vitro antiviral susceptibility profiles. Resistance to the adamantane antivirals (amantadine, rimantadine) is widespread among clade 2.1 and clade 1 H5N1 viruses [4]. Decreased susceptibility to oseltamivir was identified in viral isolates from patients with H5N1 clade 2.3.4 and clade 2.2 before antiviral treatment was initiated, suggesting that strains circulating among poultry had reduced oseltamivir susceptibility [46],[47]. It is unknown whether higher dosing has clinical effectiveness against H5N1 virus strains exhibiting reduced in vitro oseltamivir susceptibility. Development of resistance to H5N1 virus during oseltamivir treatment has been shown in clade 1 virus strains, including during early treatment [48], and was reported in a patient who had received oseltamivir chemoprophylaxis [49].

While human data on combination antiviral treatment are very limited [50], animal data support a benefit of combination antiviral treatment over monotherapy for H5N1 virus infection [23],[24]. Indeed, WHO recommended consideration of combination antiviral treatment with an adamantane plus a neuraminidase inhibitor for H5N1 patients infected with susceptible virus strains [4],[22].

The biggest challenge for physicians to initiating antiviral treatment is to identify H5N1 virus-infected patients early, before severe disease progression has occurred. Nonspecific signs and symptoms hinder clinical recognition of early H5N1 disease in most patients [4],[51], and some H5N1 patients do not always have identified exposure to H5N1 virus [44],[52]. Fortunately, H5N1 virus infection remains rare worldwide, so the only way to detect early infection is to test a huge number of suspected cases early, in which very few will have H5N1 virus infection [53]. Additionally, wide availability of an accurate, simple, inexpensive, rapid, point of care test (which does not exist currently), as well as antiviral medications, would be needed at health care facilities in countries with H5N1 poultry outbreaks. However, H5N1 virus may not always be detectable in an upper respiratory tract specimen from an infected patient during early illness [4].

Currently, most H5N1 virus-infected patients are identified and hospitalized about four to six days after illness onset when they have severe disease [4],[44],[45],[50],[54]. Given that the pathogenesis appears to include high H5N1 viral replication in the lower respiratory tract driving cytokine dysregulation [4],[6], other therapy besides late antiviral treatment or higher dosing may be needed. H5N1 patients with diarrhea may require higher dosing of antivirals, and documentation of viral dissemination (viremia, cerebrospinal fluid, brain, intestinal tract) in fatal cases suggests that intravenous treatment may be needed [4],[6],[55]. High-dose oseltamivir administration via oral gastric tube has been shown to achieve high plasma levels in two ventilated patients with H5N1 [56].

In a very small number of severely ill patients with H5N1, antivirals and immunotherapy were administered [50],[57],[58]. The source of the immunotherapy was convalescent plasma from patients with H5N1 who survived or from a participant in an H5N1 vaccine clinical trial [50],[57],[58]. All three severely ill immunotherapy recipients recovered fully. While these results are compelling, this immunotherapy was uncontrolled, few patients were treated, and other therapies, including antivirals, were administered. Clearly, additional research on such therapy is needed. But these initial results do raise questions about whether even in severely ill patients, antiviral treatment and administration of neutralizing antibodies can decrease H5N1 viral load rapidly and dampen cytokine dysregulation, allowing pulmonary recovery.

Clinical care of patients with H5N1 entails much more than antiviral treatment. Management of complications such as acute respiratory distress syndrome, hypoxemia, pleural effusions, pneumothoraces, disseminated intravascular coagulation, renal dysfunction, and multi-organ disease requires excellent intensive care [4],[51]. It is possible that improving and standardizing optimal intensive care unit (ICU) care for patients with H5N1, including ensuring adequate oxygen delivery and optimizing ventilator management, might result in lower mortality—even among patients who are admitted with severe disease. Collection of comprehensive clinical data detailing how patients with H5N1 are cared for, and training in standard ICU care and ventilator management of patients with H5N1 for clinicians in H5N1-affected countries, might lead to improved clinical management with higher patient survival. Infection control must be emphasized among health care workers and family caregivers [59]–[61]. In the setting of a disease with very high mortality, with no available controlled human clinical data to guide clinicians, in which most patients present with severe disease, a number of combined strategies should be considered for therapy of H5N1 patients. These include both pharmacological strategies (combination antiviral treatment, anti-inflammatory agents, and immunotherapy), and non-pharmacological strategies (standardization of optimal ventilator and fluid management, especially for acute respiratory distress syndrome, and management of other complications). Collection of detailed clinical data is needed to inform optimal management of patients with H5N1, with defined clinical and virological outcomes. Similar issues confront clinicians treating patients hospitalized with severe lower respiratory tract disease due to novel swine-origin influenza A (H1N1) virus infection (currently resistant to adamantane antivirals) [62].

## Abbreviations

HPAI: highly pathogenic avian influenza A
RCT: randomised clinical trial
SARS: severe acute respiratory syndrome
SARS-CoV: SARS-associated coronavirus
WHO: World Health Organization

## References

[1] World Health Organization (2003). Guidelines for WHO guidelines. EIP/GPE/EQC/2003.1.. http://www.gradeworkinggroup.org/publications/.

[2] Shekelle P, Woolf S, Eccles M, Grimshaw J (1999). Clinical guidelines: Developing guidelines.. BMJ.

[3] BBC News (2008 August 8). Flu pandemic “gravest risk to UK”.. http://news.bbc.co.uk/1/hi/uk_politics/7548593.stm.

[4] Abdel-Ghafar AN, Chotpitayasunondh T, Gao Z, Hayden FG, Writing Committee of the Second World Health Organization Consultation on Clinical Aspects of Human Infection with Avian Influenza A (H5N1) Virus (2008). Update on avian influenza A (H5N1) virus infection in humans.. N Engl J Med.

[5] Crusat M, de Jong MD (2007). Neuraminidase inhibitors and their role in avian and pandemic influenza.. Antivir Ther.

[6] de Jong MD, Simmons CP, Thanh TT, Hien VM, Smith GJ (2006). Fatal outcome of human influenza A (H5N1) is associated with high viral load and hypercytokinemia.. Nat Med.

[7] Yen HL, Monto AS, Webster RG, Govorkova EA (2005). Virulence may determine the necessary duration and dosage of oseltamivir treatment for highly pathogenic A/Vietnam/1203/04 influenza virus in mice.. J Infect Dis.

[8] Govorkova EA, Ilyushina NA, Boltz DA, Douglas A, Yilmaz N (2007). Efficacy of oseltamivir therapy in ferrets inoculated with different clades of H5N1 influenza virus.. Antimicrob Agents Chemother.

[9] Schünemann HJ, Hill SR, Kakad M, Bellamy R, Uyeki TM (2007). WHO Rapid Advice Guidelines for pharmacological management of sporadic human infection with avian influenza A (H5N1) virus.. Lancet Infect Dis.

[10] Schünemann HJ, Hill SR, Kakad M, Vist GE, Bellamy R (2007). Transparent development of the WHO rapid advice guidelines.. PLoS Med.

[11] Hayden FG, Treanor JJ, Fritz RS, Hayden FG, Treanor JJ (1999). Use of the oral neuraminidase inhibitor oseltamivir in experimental human influenza: Randomized controlled trials for prevention and treatment.. JAMA.

[12] Wattanagoon Y, Stepniewska K, Lindegårdh N, Pukrittayakamee S, Silachamroon U (2009). Pharmacokinetics of high dose oseltamivir in healthy volunteers.. Antimicrob Agents Chemother.

[13] Guyatt GH, Oxman AD, Kunz R, Falck-Ytter Y, Vist GE (2008). Rating quality of evidence and strength of recommendations: Going from evidence to recommendations.. BMJ.

[14] Guyatt GH, Oxman AD, Vist G, Kunz R, Falck-Ytter Y (2008). Rating quality of evidence and strength of recommendations GRADE: An emerging consensus on rating quality of evidence and strength of recommendations.. BMJ.

[15] Rawlins M (2009). De testimonio: On the evidence for decisions about the use of therapeutic interventions.. Lancet.

[16] Lackenby A, Hungnes O, Dudman SG, Meijer A, Paget WJ (2008). Emergence of resistance to oseltamivir among influenza A (H1N1) viruses in Europe.. Eurosurveillance.

[17] World Health Organization (2009). Influenza A (H1N1) virus resistance to oseltamivir.. http://www.who.int/csr/disease/influenza/h1n1_table/en/index.html.

[18] Clavel F, Hance AJ (2004). HIV drug resistance.. N Engl J Med.

[19] Hirsch HH, Kaufmann G, Sendi P, Battegey M (2004). Immune reconstitution in HIV-infected patients.. Clin Infect Dis.

[20] Kuritzkes DR, Walker BD, Knipe DM, Howley PM (2007). HIV-1: Pathogenesis, clinical manifestations, and treatment.. Field's virology. 5th edition.

[21] World Health Organization (2007). Continuing progress towards a unified nomenclature system for the highly pathogenic H5N1 avian influenza viruses.. http://www.who.int/csr/disease/avian_influenza/guidelines/nomenclature/en/index.html.

[22] World Health Organization (2007). Clinical management of human infection with avian influenza A (H5N1) virus. Updated advice 15 August 2007.. http://www.who.int/csr/disease/avian_influenza/guidelines/ClinicalManagement07.pdf.

[23] Ilyushina NA, Hoffmann E, Salomon R, Webster RG, Govorkova EA (2007). Amantadine-oseltamivir combination therapy for H5N1 influenza virus infection in mice.. Antivir Ther.

[24] Ilyushina NA, Hay A, Yilmaz N, Boon AC, Webster RG (2008). Oseltamivir-ribavirin combination therapy for highly pathogenic H5N1 influenza virus infection in mice.. Antimicrob Agents Chemother.

[25] Zheng BJ, Chan KW, Lin YP, Zhao GY, Chan C (2008). Delayed antiviral plus immunomodulator treatment still reduces mortality in mice infected by high inoculum of influenza A/H5N1 virus.. Proc Natl Acad Sci U S A.

[26] Aldridge JR, Moseley CE, Boltz DA, Negovetich NJ, Reynolds C (2009). From the Cover: TNF/iNOS-producing dendritic cells are the necessary evil of lethal influenza virus infection.. Proc Natl Acad Sci U S A.

[27] Hanson BJ, Boon AC, Lim AP, Webb A, Ooi EE (2006). Passive immunoprophylaxis and therapy with humanized monoclonal antibody specific for influenza A H5 hemagglutinin in mice.. Respir Res.

[28] Simmons CP, Bernasconi NL, Suguitan AL, Mills K, Ward JM (2007). Prophylactic and therapeutic efficacy of human monoclonal antibodies against H5N1 influenza.. PLoS Med.

[29] Kashyap AK, Steel J, Oner AF, Dillon MA, Swale RE (2008). Combinatorial antibody libraries from survivors of the Turkish H5N1 avian influenza outbreak reveal virus neutralization strategies.. Proc Natl Acad Sci U S A.

[30] Stockman LJ, Bellamy R, Garner P (2006). SARS: Systematic review of treatment effects.. PLoS Med.

[31] Muller MP, Dresser L, Raboud J, McGeer A, Rea E (2007). Adverse events associated with high-dose ribavirin: Evidence from the Toronto outbreak of severe acute respiratory syndrome.. Pharmacotherapy.

[32] Griffith JF, Antonio GE, Kumta SM, Hui DS, Wong JK (2005). Osteonecrosis of hip and knee in patients with severe acute respiratory syndrome treated with steroids.. Radiology.

[33] Hong N, Du XK (2004). Avascular necrosis of bone in severe acute respiratory syndrome.. Clin Radiol.

[34] Muller MP, McGeer A, Straus SE, Hawryluck L, Gold WL (2004). Clinical trials and novel pathogens: Lessons learned from SARS.. Emerg Infect Dis.

[35] Chan PK (2002). Outbreak of avian influenza A(H5N1) virus infection in Hong Kong in 1997.. Clin Infect Dis.

[36] Peiris JS, Yu WC, Leung CW, Cheung CY, Ng WF (2004). Re-emergence of fatal human influenza A subtype H5N1 disease.. Lancet.

[37] World Health Organization (2009). Cumulative number of confirmed human cases of avian influenza A/(H5N1) reported to WHO.. http://www.who.int/csr/disease/avian_influenza/country/cases_table_2009_05_15/en/index.html.

[38] Matheson NJ, Harnden AR, Perera R, Sheikh A, Symmonds-Abrahams M (2007). Neuraminidase inhibitors for preventing and treating influenza in children.. Cochrane Database Syst Rev.

[39] Jefferson TO, Demicheli V, Di Pietrantonj C, Jones M, Rivetti D (2006). Neuraminidase inhibitors for preventing and treating influenza in healthy adults.. Cochrane Database Syst Rev.

[40] Cooper NJ, Sutton AJ, Abrams KR, Wailoo A, Turner D (2003). Effectiveness of neuraminidase inhibitors in treatment and prevention of influenza A and B: Systematic review and meta-analyses of randomised controlled trials.. BMJ.

[41] Harper SA, Bradley JS, Englund JA, File TM, Gravenstein S (2009). Seasonal influenza in adults and children—Diagnosis, treatment, chemoprophylaxis, and institutional outbreak management: Clinical practice guidelines of the Infectious Diseases Society of America.. Clin Infect Dis.

[42] McGeer A, Green KA, Plevneshi A, Shigayeva A, Siddiqi N (2007). Antiviral therapy and outcomes of influenza requiring hospitalization in Ontario, Canada.. Clin Infect Dis.

[43] Lee N, Chan PK, Choi KW, Lui G, Wong B (2007). Factors associated with early hospital discharge of adult influenza patients.. Antivir Ther.

[44] Kandun IN, Tresnaningsih E, Purba WH, Lee V, Samaan G (2008). Factors associated with case fatality of human H5N1 virus infections in Indonesia: A case series.. Lancet.

[45] Liem NT, Tung CV, Hien ND, Hien TT, Chau NQ (2009). Clinical features of human influenza A (H5N1) infection in Vietnam: 2004–2006.. Clin Infect Dis.

[46] Le MTQ, Wertheim HFL, Nguyen HD, Taylor W, Hoang PVM (2008). Influenza A H5N1 clade 2.3.4 virus with a different antiviral susceptibility profile replaced clade 1 virus in humans in northern Vietnam.. PLoS ONE.

[47] Saad MD, Boynton BR, Earhart KC, Mansour MM, Niman HL (2007). Detection of oseltamivir resistance mutation N294S in humans with influenza A H5N1 [Abstract P909]..

[48] de Jong MD, Tran TT, Truong HK, Vo MH, Smith GJ (2005). Oseltamivir resistance during treatment of influenza A (H5N1) infection.. N Engl J Med.

[49] Le QM, Kiso M, Someya K, Sakai YT, Nguyen TH (2005). Avian flu: Isolation of drug-resistant H5N1 virus.. Nature.

[50] Yu H, Gao Z, Feng Z, Shu Y, Xiang N (2008). Clinical characteristics of 26 human cases of highly pathogenic avian influenza A (H5N1) virus infection in China.. PLoS ONE.

[51] Uyeki TM (2009). Human infection with highly pathogenic avian influenza A (H5N1) virus. Clinical issues.. Clinical Infect Dis.

[52] Sedyaningsih ER, Isfandari S, Setiawaty V, Rifati L, Harun S (2007). Epidemiology of cases of H5N1 virus infection in Indonesia, July 2005–June 2006.. J Infect Dis.

[53] Kitphati R, Apisarnthanarak A, Chittaganpitch M, Tawatsupha P, Auwanit W (2008). A nationally coordinated laboratory system for human avian influenza A (H5N1) in Thailand: Program design, analysis, and evaluation.. Clin Infect Dis.

[54] [No authors listed] (2006). Epidemiology of WHO-confirmed human cases of avian influenza A(H5N1) infection.. Wkly Epidemiol Rec.

[55] Gu J, Xie Z, Gao Z, Liu J, Korteweg C (2007). H5N1 infection of the respiratory tract and beyond: A molecular pathology study.. Lancet.

[56] Taylor WRJ, Thinh BN, Anh GT, Horby P, Wertheim H (2008). Oseltamivir is adequately absorbed following nasogastric administration to adult patients with severe H5N1 influenza.. PLoS ONE.

[57] Zhou B, Zhong N, Guan Y (2007). Treatment with convalescent plasma for influenza A (H5N1) infection.. N Engl J Med.

[58] Wang H, Feng Z, Shu Y, Yu H, Zhou L (2008). Probable limited person-to-person transmission of highly pathogenic avian influenza A (H5N1) virus in China.. Lancet.

[59] World Health Organization (2008). Infection control recommendations for avian influenza in health-care facilities. Aide-Memoire.. http://www.who.int/csr/disease/avian_influenza/guidelines/EPR_AM1_E5.pdf.

[60] World Health Organization (2008). Early recognition, reporting and infection control management acute respiratory diseases of potential international concern. Aide-Memoire.. http://www.who.int/csr/disease/avian_influenza/guidelines/EPR_AM4_E3.pdf.

[61] Centers for Disease Control and Prevention (2004). Interim recommendations for infection control in health-care facilities caring for patients with known or suspected avian influenza.. http://www.cdc.gov/flu/avian/professional/pdf/infectcontrol.pdf.

[62] Novel Swine-Origin Influenza A (H1N1) Virus Investigation Team (2009). Emergence of a novel swine-origin influenza A (H1N1) virus in humans.. Engl J Med.

